# Genome Sequence of *Microbacterium* sp. Strain R1, Isolated from a *Synechococcus* Culture

**DOI:** 10.1128/MRA.00542-21

**Published:** 2021-09-09

**Authors:** Ruiyu Zhou, Binbin Liu, Jihua Liu, Rui Wang

**Affiliations:** a Institute of Marine Science and Technology, Shandong University, Qingdao, People’s Republic of China; Montana State University

## Abstract

*Synechococcus* cultures in the laboratory are often associated with heterotrophic bacteria. Here, we report the genome sequence of the bacterium *Microbacterium* sp. strain R1, isolated from a culture of the estuarine *Synechococcus* strain CBW1107. Several secondary metabolites and transporter-related genes were identified in the genome of *Microbacterium* sp. strain R1.

## ANNOUNCEMENT

*Synechococcus* spp. are important primary producers in the marine environment, and heterotrophic bacteria can utilize dissolved organic matter released from *Synechococcus* spp. in the surrounding waters (1). Meanwhile, *Synechococcus* spp. also depend on the remineralization of dissolved organic matter (DOM) by heterotrophic bacteria to provide essential nutrients (2, 3). Therefore, interactions between *Synechococcus* and associated bacteria play important roles in the ocean biogeochemical cycle (4). The heterotrophic bacterial strain R1 was isolated from a culture of *Synechococcus* sp. strain CBW1107 (5). To obtain strain R1, CBW1107 was grown in SN15 medium (6) to the exponential phase; then, 100 μl of the algal solution was spread onto agar rich organic (RO) plates and incubated at 28°C for 1 to 2 days. A single colony was transferred to a new agar plate for further purification. Phylogenetic analysis based on 16S rRNA gene sequencing revealed that strain R1 belongs to the genus *Microbacterium*, which is widely distributed in both seawater and deep-sea sediment (7, 8). Here, we present the draft genome sequence of *Microbacterium* sp. strain R1.

*Microbacterium* sp. strain R1 was grown in RO medium at 28°C to the exponential phase, and bacterial cells were collected by centrifugation (10,000 × *g*, 10 min); then, bacterial genomic DNA was obtained from the cells following the phenol-chloroform method (9). A library was prepared using the TruSeq DNA library prep kit (Illumina, San Diego, CA, USA). Whole-genome sequencing was performed on the Illumina HiSeq 4000 sequencing system using HiSeq 3000/4000 sequencing-by-synthesis (SBS) kits (Illumina), following the format for an average 2 × 250-bp paired-end library. Low-quality (score, ≤20) raw reads, N nucleotide-containing reads, and filtered sequences less than 100 bp were removed to obtain clean reads using Trimmomatic V0.33 software. A total of 2,972.45 Mbp reads (clean data) were assembled using the IDBA algorithm (10). The assembled draft genome sequence has a total size of 3,986,645 bp in 21 contigs and a GC content of 68.20%. The *N*_50_ value and the average coverage (BBMap V38.92) for the contigs are 2,408,454 bp and 967.483×, respectively. Open reading frame (ORF) prediction and genome annotation were performed using the RAST server (http://rast.nmpdr.org) (11). A total of 3,929 protein-coding genes, 47 tRNA-coding genes, and 4 rRNA genes were predicted in the draft genome of *Microbacterium* sp. strain R1. Of the 3,929 ORFs, 2,369 (60.30%) have predicted functions. Default parameters were used for all software unless otherwise noted.

Potential secondary metabolites of *Microbacterium* sp. strain R1 were predicted using antiSMASH bacterial version. Four genes encoding nonribosomal peptide synthase, polyketide synthase type III, terpenes, and β-lactones were identified (12, 13). The genome of this strain contains 278 genes related to ABC transporters.

### Data availability.

The genome sequence of *Microbacterium* sp. strain R1 has been deposited in GenBank under accession no. JADDUD000000000, where the Prokaryotic Genome Annotation Pipeline (PGAP) annotation is also available. The raw sequence data are available under SRA accession no. SRR14683051. The BioProject accession no. is PRJNA667551, and the BioSample accession no. is SAMN16377710.
